# Feeding Corn Oil in a Nanoemulsified Form Alters the Unsaturated Fatty Acids in the Milk of Zaraibi Dairy Goats

**DOI:** 10.3390/ani12192559

**Published:** 2022-09-25

**Authors:** Mahmoud Atef Yousef, Mohammed Hamdy Farouk, Hossam H. Azzaz, Mostafa S. A. Khattab, Ahmed M. Abd El Tawab, Mohamed El-Sherbiny

**Affiliations:** 1 Animal Production Department, Faculty of Agriculture, Al-Azhar University, Cairo 11884, Egypt; 2 Department of Dairy Science, National Research Centre, 33 Bohouth St., Dokki, Giza 12622, Egypt

**Keywords:** nanoemulsion, corn oil, dairy goats, milk, amino acid, fatty acid

## Abstract

**Simple Summary:**

Increasing the polyunsaturated fatty acid content of ruminant milk represents a much needed step to increase the functional properties of the milk. However, boosting milk fatty acids through feeding strategies has remained a significant challenge for years; it requires new solutions to deliver unsaturated fatty acids in a much safer form for rumen microorganisms than the traditional supplemented raw oil form. The goal is to target less biohydrogenation, which results in less saturated fatty acid accumulation in the rumen and milk. In the present study, the ultrasonic nanoemulsification of corn oil was introduced as a replacement for the raw form of oil supplementation; it was used at 3% of the offered feed dry matter in a trial on dairy goats. The new form of corn oil supplementation was more effective than the raw form of corn oil in increasing milk productivity and fat percentage and preserving a more significant proportion of polyunsaturated fatty acids in the milk of dairy goats. Conversely, the raw form of corn oil resulted in milk fat depression and lower total solid content in addition to milk with higher proportions of saturated fatty acid.

**Abstract:**

Oil in water nanoemulsion represents a new and unstudied form of oil supplementation to the ruminant diet; that is why the aim was to evaluate the potential of nanoemulsified corn oil in dairy goats’ diets on milk productivity and fatty acid proportion. Twenty-four lactating Zaraibi goats in early lactation were randomly allocated to the following treatments: control—a basal diet without any supplementation, CO—the control diet + corn oil supplied at 3% on a dry matter basis (DM), NCO—the control diet + nanoemulsified corn oil provided at 3% on a DM basis. A completely randomized design that lasted 30 days (25 days of adaptation + 5 days of sampling) was used with eight goats in each treatment. The control diet consisted of 50% concentrate and 50% Egyptian berseem clover. The NCO increased the milk production, fat percentage, and yield compared to the CO and the control. The proportions of oleic, linoleic, and linolenic acids were higher in the NCO compared to the control and CO. The NCO had less effect on the biohydrogenation intermediates’ profile than the CO; noticeably, higher proportions of unsaturated fatty acid (UFA) were associated with the NCO. In conclusion, the NCO increased milk production and decreased the transformation rate of UFA to saturated fatty acids in the biohydrogenation environment.

## 1. Introduction

Feeds supplemented with polyunsaturated-fatty-acid (PUFA)-rich oils of either plant or marine origin are widely used in ruminant nutrition [1]. Previous studies showed that PUFA-rich oils alter the rumen fatty acid content, especially the accumulation of long-chain fatty acid and the formation of trans-vaccenic acid and conjugated linoleic acid, by affecting the number and activity of rumen microorganisms, mainly biohydrogenation bacteria such as *Butyrivibrio fibrisolvens* and *Butyrivibrio proteoclasticus* [1,2,3]. However, rumen lipolysis and biohydrogenation decrease the amount of dietary PUFA reaching and incorporating into ruminant products (meat and milk) [3]. Rumen microorganisms biohydrogenate dietary unsaturated fatty acids (UFAs) to saturated fatty acids (SFAs) to protect themselves from the harmful effect of the UFAs on their cell construction, especially PUFAs, which can disrupt the membrane integrity of the rumen biohydrogenation bacteria [4,5]. These processes eventually lead to an increase in the outflow of saturated fatty acid to post-ruminal digestion and absorption [4,5,6]. That is why, according to O’Donnell [7], fat from dairy products provides around 25–35% of the total intake of saturated fatty acids, while contributing up to 15–25% of the total fat humans consume daily. Based on the literature, the fatty acid composition of the milk fat from the milk of ruminants is about 70% saturated fatty acids (SFAs), 8% trans fatty acids, and less than 5% long-chain unsaturated fatty acids (LCUFAs) [7]. The intake of saturated fatty acids has been linked to increased risks of cardiovascular diseases, obesity, and arteriosclerosis [5,8]. On the other hand, LCUFAs, especially Omega 3, Omega 6, and conjugated linoleic acid (CLA), are known for their significant importance as active dietary compounds for preventing arteriosclerosis, coronary heart disease, and inflammatory conditions and retarding the growth of tumor cells based on the results of studies conducted on different animal models (including mice, rats, and pigs) [8,9]. As for children, studies have shown that docosahexaenoic acid and other Omega 3 s are essential for infant brain, eye, and nervous system development and have been shown to support long-term heart health, which was supported by trials on mice and rats and clinical studies on children [9,10]. That is why it is desirable to investigate other forms of supplemented oils for the ruminant diet that could preserve the PUFAs from being digested through rumen lipolysis and biohydrogenation, significantly without a negative effect on the rumen fermentation pattern and the rumen microorganisms at the same time [3,4,5,6]. Oil in water nanoemulsion is considered an unstudied form in ruminant nutrition; it is one of the most important nanotechnologies widely used in several scientific and practical applications. Nanoemulsions are known as multiphase colloidal dispersions formed by dispersing one liquid in another immiscible liquid by physical-share-induced rupturing at the nanoscale with droplet sizes less than 100 nm [11]. Based on previous in vitro studies [12,13], a higher outflow of unsaturated fatty acids from the rumen (bypassing the rumen) may be expected when introducing a nanoemulsified form of edible corn oil to lactating goat nutrition; hence, it may increase the unsaturated fatty acids’ profile in the produced milk. Accordingly, the hypothesis-driven idea is that the small size of dietary edible oil droplets (nanoscale) produced with nanoemulsion technology and supplied to goats’ daily nutrition will have a lower impact on the rumen fermentation and microorganisms than raw oils. Consequently, the following study aimed to evaluate the effects of corn oil in raw form (CO) or nanoemulsified form (NCO) on the productive performance (milk production and composition) and fatty acids’ profile in the milk of Zaraibi dairy goats. 

## 2. Materials and Methods

Corn oil in raw form (CO) and nanoemulsified form (NCO) was evaluated in a trial using twenty-four lactating Zaraibi goats to study its effect on goats’ productive performance (milk production and composition), milk amino acids’ profile, and fatty acids’ proportions. The corn oil was chosen based on an earlier in vitro evaluation phase of different oil nanoemulsions produced from three edible oils (olive oil, corn oil, and linseed oil) purchased from local markets in Egypt; these studies showed that corn oil at 3% of supplementation had positive effects on the rumen volatile fatty acid formation and the PUFA accumulation when compared to nanoemulsions produced from olive oil and linseed oil.

### 2.1. Locations of the Study

The trial on dairy Zaraibi goats was conducted at the experimental farm of the Faculty of Agriculture, Al-Azhar University, Cairo, Egypt (30°03′29.7″ N 31°18′45.1″ E). The dairy goats’ experimentation, handling, and management followed the guidelines of the Polish act on the protection of animals used for scientific and educational purposes (Dz.U. 26 January 2015, item 266), authorized by the granting committees of the Science, Technology, and Innovation Funding Authority (STDF) and the National Research Centre, Egypt. The experiment was also authorized by the Faculty of Agriculture, Al-Azhar University, Egypt. Nanoemulsified corn oil preparation, chemical analysis of the collected samples, and related data management, processing, and manuscript preparation were performed at the Laboratory of Dairy Production, Department of Dairy Science, National Research Centre, Egypt.

### 2.2. Nanoemulsions Preparation

Corn oil in water emulsion was premixed at 13,500 rpm with a digital high-speed homogenizer for 2 min (HG-15D Homogenizer, Daihan Scientific C., Gangwon-Do, South Korea) to reach a droplet size distribution of 5.3 ± 0.6 μm. Then, the preparation of corn oil in water nanoemulsion was performed on the pre-homogenized solution using a Sonics VCX750 ultrasonic processor with 750-Watt nominal power and a frequency of 20 kHz equipped with a 25 mm sonotrode tip (80% amplitude for 20 min; Sonics and Materials, Newtown, USA). which resulted in a droplet size distribution range of 25–190 nm, where 90% of that distribution was on average 58 ± 11 nm. The procedure stated was conducted according to the findings of [14]. The oil in water emulsion formulation was 15% corn oil (purchased from the Egyptian market), 5.6% Tween 80 (Sigma Aldrich, Darmstadt, Germany), and 79.4% distilled water. A Zetasizer Nano ZS (Malvern Instruments, United Kingdom) was used to track the size of the produced nanoemulsion liquid suspension measured weekly at 25 °C.

### 2.3. Animal Management

In a completely randomized design with a 25-day adaptation period and a 5-day sampling period for a total of 30 days, 24 lactating Zaraibi goats (33 ± 1.5 kg body weight, 2.6 ± 0.7 parity (the parity per treatment was as follows: control 2.7 ± 0.7, raw corn oil 2.6 ± 0.7, and nanoemulsified corn oil 2.5 ± 0.6), 7 ± 2 d in milk, 32 ± 5.3 months of age, and 812 ± 55 g/d of milk production in the previous lactation season (mean ± SD)) were used. The goats in each group were kept with their kids in separate stalls (7 × 10 m each), and they were fed in treatment groups based on body weight. According to the NRC [15], the supplied feed was designed to suit the nutritional needs of a mature dairy goat with twin kids and body weight between 30 and 40 kg (all goats assigned to the experiment had twin kids).

### 2.4. Diet and Treatments

The offered feed was composed of 50% concentrate feed mixture and 50% roughage (berseem clover), as shown in Table 1. Initially, after one week of parturition, the lactating goats were randomly allocated (8 goats per group) to one of the following treatments: control—a basal diet without any supplementation consisting of a 50:50 concentrate to roughage ratio as described in Table 1: CO—the control group diet + 3% of corn oil in the raw form calculated on the DM basis, and NCO—the control group diet + 3% of corn oil in the nanoemulsified form calculated on the DM basis. The corn oil supplementation level and type were selected based on the in vitro results obtained from previous studies. Feed was offered to lactating goats twice daily at 7 a.m. and 7 p.m. The feed used for all treatments was analyzed in triplicate every ten days to check the total chemical composition, especially dry matter, and adjust it if necessary. The raw corn oil supplementation (CO) was mixed with 150 g of concentrate and then offered individually for each animal twice daily before the morning and evening feeding to ensure that each animal received the specified amount of oil supplementation. However, the nanoemulsified form of corn oil (NCO), prepared daily, was divided into four equal portions, then offered to the goats to drink (using a 50 mL plastic syringe) at an interval of three hours from 7 a.m. to 7 p.m. Samples from the feed and milk of the lactating goats were collected during the sampling period. The fatty acid composition of the offered feed and supplements is shown in Table 2.

### 2.5. Monitoring Production and Sampling

During the sampling period, does were separated from their kids for 12 h from 20:00 to 08:00 of the following day to record the milk production yield, and all does were hand-milked at 08:00 on Days 26 to 30 of the experiment. After milk recording, two aliquots of milk (50 mL each) were sampled from all goats throughout the sampling period (for each animal: 1 time-point × 2 tubes (50 mL) × 5 days). One of the collected milk aliquots was immediately subjected to an infrared examination to determine the chemical makeup of the milk; however, the second aliquot was stored at −20 °C for a future chromatographic study (amino acid and fatty acid). Every ten days, a sample of the fresh feed was taken and kept at −20 °C for chemical analysis. On Days 26, 28, and 30 of the experiment, 10 mL of blood samples was collected from all goats at 4 h after the morning feeding from the jugular vein. Blood samples in tubes without anticoagulants were centrifuged at 5000× g for 15 min at 4 °C, and the serum was collected into 2 mL Eppendorf tubes and frozen at −20 °C.

### 2.6. Chemical Analysis

The feed samples (concentrate and berseem) collected every 10 days were dried separately for 48 h at 55 °C, then milled to pass through a 1 mm screen (FZ102, Shanghai-Hong Ji Instrument Co., Ltd., Shanghai, China). Ground dry samples were analyzed separately in triplicate for the dry matter content, ash, crude protein, and ether extract; following each corresponding official method of the AOAC [16], the organic matter (OM) was calculated by the difference. Neutral detergent fiber (NDF) and acid detergent fiber (ADF) analyses were performed using an ANKOM200 Fiber Analyzer unit (ANKOM Technology Corporation, Macedon, NY, USA) following the method described in [16,17] and expressed without residual ash. Samples analyzed for NDF were pretreated with alpha-amylase and sodium sulfite at the start of the analysis. Milk samples collected daily (from Day 26 to Day 30 of the experiment) for the basic chemical composition were analyzed in triplicate for fat, crude protein, and lactose using a milk analyzer equipped with an infrared measuring instrument (Milkotester LM2, Belovo, Bulgaria). The milk total and non-fat solids content was calculated. The collected blood serum was analyzed for total proteins, albumin, urea-N, glucose, alanine aminotransferase (ALT), aspartate aminotransferase (AST), triglycerides, high-density lipoprotein cholesterol (HDL), low-density lipoprotein cholesterol (LDL), beta-hydroxybutyrate (BHB), and non-esterified fatty acids (NEFAs) using test-specific ready kits (Cayman Chemical, Ann Arbor, MI, USA). The globulin concentration was calculated by subtracting the albumin concentration from the total protein concentration.

Quantitative amino acid (AA) measurements were performed for the milk protein in accordance with Millipore Corporation’s HPLC-Pico-Tag method following the preparation process [18]. Briefly, phenyl isothiocyanate (PITC, also known as Edman’s reagent) was utilized for pre-column derivatization. At the same time, phenylthiocarbamide (PTC) derivatives identified by their UV absorbance were separated using reversed-phase gradient elution high-performance liquid chromatography (HPLC). The sample corresponding to the protein was weighed into a 25 × 150 mm hydrolysis tube and placed in an oven for 24 h at 110 °C. Quantitatively, the tube contents were transferred to a volumetric flask, and the desired volume was then achieved using HPLC-grade water. A 0.45 µm sample filter was used to filter around 1 mL of the solution. Aliquots of the hydrolysate were poured into a reaction vial with the proper standards in soda glass from Fisons and disposable glass sample tubes from Waters Associates. After attaching the vial to the workstation manifold and opening the vacuum control valve, the samples were dried under vacuum in a PICO-Tag workstation to eliminate the hydrochloric acid. The process of derivatization was started by adding a freshly made reagent, mixing it with a vortex, and letting it sit at room temperature for 20 minutes. Derivatized samples can be kept in this dried state for several weeks in the freezer before analysis, if necessary. The gradient of the Pico-Tag solvent (Eluents A and B) at 38 °C and a flow rate 1 mL/min was followed for HPLC analysis, and 20 mL of the sample was injected and loaded on an amino acid stainless steel C18 column (100 × 4.6 mm). Using UV absorption measurements with a fixed wavelength (254 nm) (Waters detector), the PTC derivatives were detected. The apparatus was calibrated using two injections of the lysine standards before injecting the samples.

Following the fatty acid methyl ester (FAME) analysis outlined in [12], the milk and dry ground feed’s fatty acid content samples were examined. Briefly, 500 mg and 100 mg of milk and dry feed samples, respectively, were introduced to 3 mL of 2 M NaOH for hydrolysis of the samples in a closed system using 15 mL screw-cap Pyrex tubes with Teflon stoppers. The hydrolyzed samples were incubated for 40 min at 90 °C in a block heater. The samples were then extracted, esterified in methanol with 0.5 M NaOH, and transformed to FAME in boron trifluoride (1.3 M; Fluka-Sigma Aldrich, St. Louis, MO, USA). A gas GC-MS system (7890B, Agilent, Santa Clara, CA, USA) with a mass spectrometer detector and a 100 m fused silica capillary column (0.25 mm i.d., coated with 0.25 m Agilent HP; Chrompack CP7420; Agilent Technologies, Santa Clara, CA, USA) (5977A) was used. During the chromatographic FAME analysis, the carrier gas was hydrogen, flowing at 1.3 mL/min. Temperatures for the injector and detector were 200 °C and 250 °C, respectively. The oven’s temperature was set to start at 120 °C for 7 min, elevating by 7 °C per minute, reaching 140 °C, held for 10 min, and then increased by 4 °C per minute to 240 °C. The sample was injected into the GC Column in a volume of 1 µL, and the peaks were detected by comparing them to the retention times of the relevant FAME standards (37 FAME Mix, Sigma Aldrich, PA, USA) using Open Lab CDS version 2.6 (Agilent, Santa Clara, CA, USA). 

Additionally, the retention time of a reference standard was compared with the conjugated linoleic acid peaks to identify them (a mixture of cis- and trans-9,11 and 10,12- octadecadienoic acid methyl esters; Sigma Aldrich, PA, USA). For the AA and FA analyses, the compositions of the two groups of acids were given as g/100 g of milk protein and total FA, respectively. The chromatographic AA and FA analyses were performed at the National Research Centre, Egypt, by the Central Service Unit.

### 2.7. Statistical Analysis

Before analysis, the results of the amino acids (AAs), fatty acids (FAs), blood chemistry, and milk production/composition were averaged by goat. The PROC MIXED procedure of SAS (SAS^®^ OnDemand for Academics, 2022 SAS Institute Inc., Cary, NC, USA) was used to analyze all parameter data (milk production/composition, milk AA, and FA profile) using a model that included the fixed effect of treatment and the random effect of the goat within the treatment. Multiple comparison Tukey tests were used to evaluate differences between treatments. At *p* ≤ 0.05 and 0.05 < *p* ≤ 0.10, treatment effects were deemed significant or trending towards significance, respectively. The means and pooled standard errors of the means are displayed for all values.

## 3. Results

### 3.1. Blood Chemistry

The effect of using corn oil in raw and nanoemulsified forms in dairy goats’ nutrition at 3% of the dry matter intake (DMI) on the blood parameters is presented in Table 3. No significant effect was observed by treatment on the blood chemistry parameters. However, the blood globulin and glucose levels tended to increase (*p* > 0.05) with the nanoemulsified corn oil supplementation (NCO) when compared to the control diet and raw corn oil treatment (CO); the NCO also tended to decrease the low-density lipoprotein cholesterol (LDL) when compared to CO.

### 3.2. Milk Composition

The effects of using corn oil in both raw form and nanoemulsified form (3% of the DMI) on milk production, yield, and composition are presented in Table 4. In the case of milk production, our hypothesis was ascertained by an increase in milk productivity (*p* < 0.05) by 21% and 25% when the dairy goats drank the nanoemulsified corn oil (NCO) compared to the control or the traditional raw corn oil supplementation (CO), respectively. The increase in productivity in the NCO was reinforced by a significant increase in fat percentage compared to the control, which was lower; however, the lowest fat % was observed in the CO group. The high fat % in the NCO also resulted in higher total solids % compared to both the CO and the control. Additionally, due to the increase in milk productivity and noticeably higher fat and protein percentage levels, the fat, protein, lactose, and energy yields were significantly higher for the NCO than those obtained in the control and CO.

### 3.3. Milk Amino Acid and Fatty Acid Proportion

The effect of nanoemulsified corn oil on the milk amino acid and fatty acid proportions in dairy goats is presented in Table 5 and Table 6, respectively. Higher proportions (*p* > 0.05) of arginine, isoleucine, valine, and alanine were observed with the NCO compared to the CO. The raw form of corn oil had a higher impact on the biohydrogenation intermediates, where we can find a significantly higher profile of vaccenic acid (TVA) and C18:2 cis-9 trans-11 when compared to both the control and NCO. On the other hand, the NCO treatment preserved (*p* < 0.05) a higher profile of oleic acid and linoleic acid compared to both the CO and control. The NCO preserved (*p* < 0.05) more monounsaturated fatty acid (MUFA) and polyunsaturated fatty acid (PUFA) when compared to the CO and control. This expected finding was also summarized by the NCO’s decrease in saturated fatty acids (SFAs) at the expense of an increase in unsaturated fatty acids (UFAs), which was noticeably higher when compared to the CO and control.

## 4. Discussion

In the last few decades, several studies have been conducted mainly to illustrate the effects of oil supplementation on milk productivity and fatty acid composition. Most of the findings emphasized the beneficial effects of additional raw forms of oils on several metrics. However, specific detrimental effects, such as the depression of milk fat, were also reported [6,19]. In the current study, nanoemulsified corn oil was introduced at a recommended level of supplementation (3% of DMI) as a potential replacement for raw oil supplementation, with the hypothesis that it would have a less detrimental effect on the rumen fermentation characteristics and a better effect on maintaining PUFA in the biohydrogenation environment and, consequently, in the final product, the milk.

### 4.1. Milk Production and Composition

According to the NRC’s [15] recommendation, the dietary fat content in the lactating ruminant diet should not exceed 6–7% of the offered dietary dry matter (DM). The total fat content in the control diet represented around 3.9% of the average DM. The level of corn oil supplementation was calculated as 3% of the dry matter; in other words, the fat level in the total diet falls under the recommended level set by the National Research Council, USA. However, supplementing corn oil in raw form seems to severely impact the milk composition. According to Zheng et al. [20], who supplemented soybean oil at 2% of the dry matter intake (DMI), reaching a level of 6–7% of ether extract in the diet, this resulted in a similar effect on milk production when compared to the control diet. However, a significant decrease in fat percentage was obtained by around 13% less than the control. A similar finding was obtained by [21,22]; a reduced fat percentage was found at 14.2% less when soybean oil was supplied at 3.4% of the DMI, with no effect on milk productivity. However, when soybean oil was supplemented at around 5% of the DMI by [23], milk fat percentage and yield reduction were noticed; however, milk production was found to be lower only in late lactating animals. In general, milk fat depression results from ineffective ruminal fermentation in the presence of high levels of PUFA [24,25]. This supports the findings of our study, in which milk fat depression (MFD) was obtained as a result of corn oil supplementation accompanied by a non-significant reduction in milk production compared to the control. The similar effect on protein and lactose percentages in our results was supported by previous research by other authors, which resulted in unaffected protein and lactose percentages when edible oils were supplemented [23,26]. This could be related to decreased fiber digestion and feed degradability when corn oil is supplemented with the dairy goat diet [27].

In the case of nanoemulsified corn oil (NCO) feeding, an adverse effect was obtained compared to those reported for raw corn oil (CO). The NCO significantly increased milk productivity; consequently, higher fat, protein, and lactose yields were observed compared to both the CO and the control. These findings are not supported by the literature, but following our previous findings [12,13], we can briefly discuss that the boost in productivity and milk composition by the NCO could be related to a lower impact on rumen fermentation and rumen microorganisms, allowing a higher amount of the nanoemulsified form to bypass the rumen to be available for absorption. This explanation could clarify the boost in the energy level supplied to the dairy goat without altering the overall performance, resulting in higher productivity and a favorable fat yield.

### 4.2. Milk Fatty Acid Composition

In the case of the fatty acid profile in the produced milk, feeding edible oils rich in unsaturated fatty acids, especially oleic and linoleic acids, is usually associated with a modulation in the unsaturated fatty acid profile and a decreased saturated fatty acid profile, except for the stearic acid level, which tends to increase with edible oil supplementation [22,23]. The fatty acids in milk usually originate from uptake from blood circulation or de novo synthesis within the mammary gland [24,28]. Unsaturated-fatty-acid-rich oil supplementations are susceptible to ruminal biohydrogenation, resulting in considerable proportions of C18:1 trans-11 and conjugated linoleic acid isomers, together with an expected increase in the C18 SFA level [29,30]. In our results, it was clear that corn oil (CO) supplementation for dairy goats’ feed resulted in a significantly higher proportion of biohydrogenation intermediates, supported by the findings of [31]. However, what we found unexpected is the high proportion of oleic acid in the CO when compared to the control group. This could result from a possible disturbed ruminal fermentation, resulting in a higher outflow of oleic acid to the small intestine. This finding is also supported by the higher proportion of the sum of UFA and the lower proportion of SFA compared to the control, in addition to the obtained MFD. In the case of NCO feeding, the resulting fatty acids seem to align with what we reported in the previous in vitro experiments [12,13]. 

The NCO seems to be able to preserve a higher level of unsaturated fatty acid from being biohydrogenated in the rumen. This came clearly with a significantly higher proportion of linoleic acid compared to the CO and the control, in addition to the lower proportion of biohydrogenation intermediates. To summarize the general effect of the NCO, we can suggest that the nanoemulsion technology changes the oils to a less toxic form for rumen microorganisms [12,13]. The possible inhibition or bypassing of ruminal lipolysis and ruminal biohydrogenation, as supported by the results of [32], in addition to the possible uptake of the rumen microorganism to the small droplet of the supplemented nanoemulsified soybean oils, as suggested by Bauchart et al. [33], could be the reason why the nanoemulsified form of corn oil acted differently when compared to the same level of raw corn oil addition. Other research highlighted that the bioaccessibility of lipids and vitamin D3 are notably higher when using nanoemulsion as a delivery system for oil-soluble vitamins [34], which can also support the idea that using corn oil in the nanoemulsified form also has better solubilization and bioaccessibility in post-ruminal digestion, leading to high incorporation of PUFAs in milk, despite the possible fact that nanoemulsion is effective in positively modulating the PUFAs in milk together with an increase in milk productivity and yields. Several studies are needed to verify the mode of action on a cellular level to track the mode of action occurring in both the rumen and blood.

## 5. Conclusions

We hypothesized a preservative action of the nanoemulsion technology against the rumen biochemical process, consequently achieving a higher flow of the unsaturated fatty acid to the produced milk. A higher milk production and fat percentage were obtained with nanoemulsified corn oil supplementation at 3% of the fed DM. Compared to the control and raw corn oil groups, increased fat, protein, lactose, and energy yields were also observed with nanoemulsion. These findings were supported by a higher proportion of PUFAs and a lower proportion of saturated fatty acids and trans C18:1 fatty acids in the milk compared to the control group. In general, the nanoemulsified form of corn oil (NCO) seems to have the ability to preserve a higher proportion of unsaturated fatty acids from being saturated in the biohydrogenation environment without negatively affecting milk production and composition. Those findings could represent a promising way to deliver edible oils as the source of unsaturated fatty acids in dairy ruminants’ nutrition. Additionally, the higher proportions of PUFAs observed with the NCO, especially Omega 3 and Omega 6 fatty acids, may promote the functional quality and health benefits of the produced milk when consumed by humans.

## Figures and Tables

**Table 1:** Ingredients and chemical composition of the control diet.

Item	Control Diet
Ingredients, g/kg of DM	
Corn grain	75.5
Cotton seed meal	116
Sunflower seed meal	85.5
Wheat bran	175
Molasses	35.5
Mineral–vitamin mixture	12.5
Berseem clover	500
Chemical composition, g/kg of DM	
Organic matter	903
Ash	97.0
Crude protein	162
Either extract	39.0
Neutral detergent fiber	365
Acid detergent fiber	205

**Table 2:** Fatty acid composition (g/100 g of FA) of the control diet and supplements.

Item	Control	Supplements ^1^	
		CO	NCO
C14:0	0.74	0.10	0.21
C16:0	21.6	12.2	9.56
C18:0	2.89	2.10	1.93
C18:1 cis-9	21.8	28.3	29.9
C18:2 cis-9,cis-12	44.9	55.1	56.3
C18:3 cis-9,cis-12,cis-15	7.71	0.90	0.82
Other FA ^2^	0.36	1.30	1.28
SFA ^3^	25.6	15.2	12.5
UFA ^4^	74.7	84.8	87.6
MUFA ^5^	52.6	28.8	30.4
PUFA ^6^	21.8	56.0	57.1

^1^ Supplements: CO—corn oil, NCO—nanoemulsified corn oil; ^2^ sum of other fatty acids including C6:0, C8:0, C10:1, C11:0, C12:0, C13:0, C19:0, C18:2 cis-9,cis-15, C21:0, C20:2, and C22:0; ^3^ sum of saturated fatty acids; ^4^ sum of unsaturated fatty acids; ^5^ sum of monounsaturated fatty acids; ^6^ sum of polyunsaturated fatty acids.

**Table 3:** Effect of the supplementation of raw and nanoemulsified corn oil on blood measurement of dairy goats.

Item ^3^	Control	CO ^1^	NCO ^2^	SEM	*p*-Value
Total proteins, g/dL	6.95	6.84	6.89	0.013	0.122
Albumin, g/dL	3.73	3.71	3.70	0.011	0.655
Globulin, g/dL	3.22	3.13	3.19	0.015	0.098
Urea-N, mg/dL	22.8	23.1	22.4	0.084	0.288
Glucose, mg/dL	63.1	63.9	65.9	0.347	0.076
ALT, units/L	14.8	14.3	14.5	0.061	0.332
AST, units/L	30.8	31.3	30.7	0.077	0.176
Triglycerides, mg/dL	180	182	187	0.867	0.110
HDL, mg/dL	88.9	89.3	89.1	0.048	0.567
LDL, mg/dL	69.7	70.1	69.1	0.121	0.089
BHB, mg/dL	0.91	0.89	0.88	0.004	0.144
NEFA, mg/dL	1.66	1.73	1.73	0.009	0.237

^1^ CO: control diet + 3% corn oil in raw form; ^2^ NCO: control diet + 3% corn oil in nanoemulsified form; ^3^ blood measurements: ALT, alanine aminotransferase; AST, aspartate aminotransferase; HDL, high-density lipoprotein cholesterol; LDL, low-density lipoprotein cholesterol; BHB, beta-hydroxybutyrate; NEFA, non-esterified fatty acid.

**Table 4:** Effect of the supplementation of raw and nanoemulsified corn oil on the milk production and composition of dairy goats.

Item	Control	CO ^1^	NCO ^2^	SEM	*p*-Value
Milk production, kg	1.22 ^b^	1.18 ^b^	1.48 ^a^	0.069	0.001
Fat %	3.99 ^b^	3.89 ^c^	4.20 ^a^	0.026	0.003
Protein %	3.35 ^b^	3.31 ^b^	3.44 ^a^	0.021	0.018
Lactose %	4.11	4.07	4.09	0.009	0.092
Total solids %	11.4 ^b^	11.3 ^b^	11.7 ^a^	0.050	0.008
Solids not fat %	7.46 ^b^	7.38 ^b^	7.53 ^a^	0.029	0.012
Energy, Mcal/kg	0.68 ^ab^	0.66 ^b^	0.70 ^a^	0.004	0.009
Fat yield, g	48.7 ^b^	45.9 ^c^	62.2 ^a^	3.199	<0.001
Protein yield, g	40.9 ^b^	39.0 ^b^	50.9 ^a^	2.592	<0.001
Lactose yield, g	50.1 ^b^	48.0 ^b^	60.5 ^a^	2.913	<0.001
Energy yield, Mcal	0.83 ^b^	0.78 ^b^	1.04 ^a^	0.053	<0.001

^a–c^ Means within a row with different superscripts differ (*p* < 0.05); ^1^ CO: control diet + 3% corn oil in raw form; ^2^ NCO: control diet + 3% corn oil in nanoemulsified form.

**Table 5:** The effect of the supplementation of raw and nanoemulsified corn oil on the amino acid composition in the milk of dairy goats.

Item	Control	CO ^1^	NCO ^2^	SEM	*p*-Value
Arginine	2.12	2.08	2.16	0.012	0.093
Histidine	2.01	2.09	2.12	0.017	0.117
Isoleucine	3.89	3.97	4.02	0.019	0.088
Leucine	8.12	8.23	8.19	0.017	0.322
Lysine	6.18	6.23	6.12	0.015	0.109
Methionine	7.12	7.35	7.23	0.035	0.216
Phenylalanine	3.93	3.99	3.89	0.015	0.143
Threonine	4.16	4.20	4.09	0.016	0.134
Valine	6.01	5.88	5.93	0.019	0.075
Alanine	3.88	3.69	3.73	0.030	0.066
Aspartic acid	5.89	6.01	5.95	0.018	0.199
Glutamic acid	19.3	19.8	19.1	0.109	0.356
Serine	4.36	4.29	4.22	0.021	0.453
Tyrosine	1.57	1.63	1.66	0.014	0.173

^1^ CO: control diet + 3% corn oil in raw form; ^2^ NCO: control diet + 3% corn oil in nanoemulsified form.

**Table 6:** The effect of the supplementation of raw and nanoemulsified corn oil on the fatty acid composition in the milk of dairy goats.

Item	Control	CO ^1^	NCO ^2^	SEM	*p*-Value
C8:0	1.54 ^b^	1.71 ^a^	1.56 ^b^	0.018	0.002
C10:0	1.93 ^a^	1.78 ^b^	1.72 ^b^	0.021	0.009
C12:0	3.35 ^a^	2.81 ^b^	2.79 ^b^	0.061	<0.001
C14:0	3.57 ^a^	2.83 ^b^	2.53 ^c^	0.103	<0.001
C14:1 cis-9	0.99 ^b^	1.29 ^a^	0.79 ^c^	0.048	0.012
C16:0	29.6 ^b^	30.3 ^a^	27.2 ^c^	0.313	<0.001
C16:1 cis-9	1.45 ^b^	1.58 ^a^	1.61 ^a^	0.016	0.008
C18:0	15.7 ^b^	17.7 ^a^	13.3 ^c^	0.424	<0.001
C18:1 trans-10	1.12 ^b^	1.51 ^a^	0.78 ^c^	0.070	0.011
C18:1 trans-11	2.19 ^b^	3.14 ^a^	1.02 ^c^	0.204	<0.001
C18:1 cis-9	21.4 ^c^	23.6 ^b^	27.5 ^a^	0.594	<0.001
C18:2 cis-9 cis-12	2.12 ^a^	2.57 ^b^	3.77 ^a^	0.164	0.003
C18:3 cis-9 cis-12 cis-15	0.69 ^b^	0.83 ^b^	1.37 ^a^	0.069	0.022
C18:2 cis-9 trans-11	0.83 ^b^	1.49 ^a^	0.63 ^a^	0.087	<0.001
C18:2 trans-10 cis-12	0.22 ^b^	0.49 ^a^	0.14 ^c^	0.035	<0.001
C20:0	0.24 ^b^	0.31 ^a^	0.23 ^b^	0.008	0.012
C22:1	0.13 ^b^	0.17 ^a^	0.12 ^b^	0.005	0.019
C20:5n-3	0.12 ^b^	0.11 ^b^	0.19 ^a^	0.008	0.021
C22:6n-3	0.09 ^b^	0.10 ^b^	0.14 ^a^	0.005	0.007
Other FA ^3^	12.7 ^a^	5.38 ^b^	12.6 ^a^	0.809	0.022
SFA ^4^	60.4 ^a^	60.1 ^a^	55.1 ^b^	0.572	<0.001
UFA ^5^	39.6 ^b^	39.9 ^b^	44.9 ^a^	0.672	<0.001
MUFA ^6^	30.1 ^c^	31.4 ^b^	33.9 ^a^	0.372	<0.001
PUFA ^7^	9.52 ^b^	8.47 ^c^	10.9 ^a^	0.243	<0.001

^a–c^ Means within a row with different superscripts differ (*p* < 0.05); ^1^ CO: control diet + 3% corn oil in raw form; ^2^ NCO: control diet + 3% corn oil in nanoemulsified form; ^3^ sum of other fatty acid including C6, C10:1, C11:0, C16:1 trans, C18:1 trans-5, C18:1 trans-9, C18:1 cis-11, C18:1 cis-12, C18:1 cis-14, C18:1 cis-15, C18:2 cis-9 cis-15, C19:0, C20:1 trans, C21:0, C20:2, C22:0, C23:0, C22:2, C24:0, and C24:1; ^4^ sum of saturated fatty acids; ^5^ sum of unsaturated fatty acids; ^6^ sum of monounsaturated fatty acids; ^7^ sum of polyunsaturated fatty acids.

## Data Availability

The data presented in this study are available upon request from the corresponding author.
